# *FATP4* Switches Cellular Lipid Utilization via the PI3K-AKT Pathway in Goat Preadipocytes

**DOI:** 10.3390/ani16081129

**Published:** 2026-04-08

**Authors:** Haiyang Li, Qi Li, Wenyang Zhang, Yuling Yang, Yong Wang, Yaqiu Lin, Zhanyu Du, Changhui Zhang, Lian Huang, Jiangjiang Zhu, Hua Xiang

**Affiliations:** 1 Qinghai-Tibetan Plateau Animal Genetic Resource Reservation and Utilization Key Laboratory of Sichuan Province, Southwest Minzu University, Chengdu 610041, China; 2 Key Laboratory of Qinghai-Tibetan Plateau Animal Genetic Resource Reservation and Utilization (Southwest Minzu University), Ministry of Education, Chengdu 610041, China; 3 Institute of Qinghai-Tibetan Plateau, Southwest Minzu University, Chengdu 610041, China

**Keywords:** IMF, *FATP4*, RNA-seq, intramuscular adipocyte, PI3k-Akt

## Abstract

The role of fatty acid transporter 4 (*FATP4*) in regulating lipid metabolism has been well studied. However, how it affects IMF deposition, especially in goats, remains poorly understood. Here, we cloned the whole coding sequence of the goat *FATP4* gene and revealed its closest affinity to sheep by amino acid sequence blast analysis. In addition, we found that the *FATP4* reached its highest expression level at day 6 of goat preadipocyte differentiation in vitro. Functionally, in cultured goat intramuscular preadipocytes, siRNA-induced *FATP4* knockdown dramatically raised the mRNA expression of lipogenesis-related genes and encouraged lipid deposition. At the same time, *FATP4* deficiency inhibited cell proliferation and significantly decreased apoptosis. Unexpectedly, although the overexpression of *FATP4* promoted cell proliferation and suppressed apoptosis, it only slightly decreased cellular lipid deposition in goat intramuscular preadipocytes. For RNA-seq (performed on pooled cell samples with three technical replicates), a total of 467 differential genes (DEGs) were identified after silencing of *FATP4* in goat preadipocytes, including 47 upregulated genes and 420 downregulated genes. These DEGs were mainly enriched in the signaling pathways of Focal adhesion, HIF-1, and PI3K-Akt by KEGG analysis. To validate these findings, knockdown of *FATP4* increased the expression of phosphatidylinositol 3-kinase (PI3k) and vice versa. Convincingly, we rescued the phenotype observed in *FATP4* knockout goat preadipocytes by blocking the PI3k-Akt signaling pathway with an AKT inhibitor (LY294002). In summary, in our in vitro model, *FATP4* plays a crucial role in directing fatty acids toward cell proliferation (prioritized over cellular lipid deposition) via the PI3K-Akt signaling pathway in goat intramuscular preadipocytes. These findings provide preliminary mechanistic insights into the regulatory network of IMF formation at the cellular level, and offer theoretical clues for future research aimed at enhancing meat quality from the standpoint of IMF deposition.

## Simple Summary

The purpose of this work was to evaluate the involvement of fatty acid transporter 4 (*FATP4*) in the control of lipid metabolism in cultured goat intramuscular preadipocytes. The entire coding sequence of the goat *FATP4* gene was cloned, its expression during the differentiation of preadipocytes was examined, and the impact of *FATP4* gene activity on fat cell formation, growth, programmed cell death, and fat accumulation in goats’ intramuscular preadipocytes was studied in vitro. Key findings from our research reveal that *FATP4* is crucial in controlling lipid use in goat preadipocytes via the PI3K-AKT signaling route, emphasizing cell growth over fat accumulation in the in vitro model. These results provide mechanistic insights into the molecular processes underlying lipid deposition in goat intramuscular preadipocytes, and offer preliminary clues for future research aiming to improve meat quality through the regulation of intramuscular fat deposition.

## 1. Introduction

With the improvement of living standards, the nutritional requirements of meat have increased unabated, and intramuscular fat (IMF) content is a very important factor for measuring the meat quality of animals [1]. IMF is the fat placed between muscle fibers and plays a critical role in enhancing the flavor, softness and juiciness of meat-based foods, hence boosting their overall taste and quality [2,3]. Therefore, elucidating the molecular mechanism of IMF deposition is of great significance for improving goat meat quality.

FATPs (fatty acid transport proteins) are a set of proteins involved in fatty acid absorption [4], largely localized inside cells and the cellular membrane, and have a crucial function in long-chain fatty acid transport [5,6]. FATPs are constituted of six isoforms that are tissue-specific and expressed by a specific gene, and they widely participate in fatty acid metabolism, cell growth, cell proliferation, and the development of various malignancies [7,8,9]. As the fourth member of FATPs, *FATP4* is widely expressed in the heart, brain, lung, liver, kidney, and small intestine [6,10], and is mainly located in the membranes of hepatocytes and small intestinal and mucosal cells. Due to its high affinity for long-chain fatty acids, *FATP4* participates in the uptake and transport of fatty acids [11]. For example, in mature enterocytes, *FATP4* was the transporter of long-chain fatty acids across the plasma membrane [12], and the silencing of *FATP4* significantly suppressed its uptake of oleate [12]. In HEK293 cells, the overexpression of *FATP4* increased the uptake of long-chain fatty acids [12]. In addition, in ETEC K88-challenged pigs, the elevated expression of *FAPT4* by KR-32 treatment increased fatty acid uptake by restoring the epithelial barrier [13]. However, in human hepatoblastoma HepG2 cells and mouse hepatocytes [14], a deficiency in *FATP4* resulted in reduced β oxidation, accompanied by increased TAG synthesis, lipoprotein breakdown and TAG breakdown. How *FATP4* affects IMF deposition in goat remains unclear.

It was also discovered that a rise in adipocyte count plays a crucial role in the formation of IMF. Silencing of *FATP4* suppressed cell proliferation in human breast cancer cells [15], mouse lung cancer cell lines [16], and mouse breast cancer cells [17]. In *FATP4*-deficient mice, it was found that the deletion of *FATP4* resulted in the proliferation of epidermal cells [18]. However, there are limited investigations on the function of *FATP4* in domestic animals. The regulating function of *FATP4* on cell proliferation during goat IMF development is unknown. Considering both the accumulation of lipids at the cellular level and the rate of cell growth could aid in comprehensively grasping *FATP4*′s function in controlling the creation of the IMF.

In this work, we investigated the effect of *FATP4* in goat intramuscular adipocytes, including cellular lipid deposition and cell proliferation, using RNA silencing and overexpression. In addition, RNA-seq was used to screen differentially expressed genes affected by loss-of-function of *FATP4* to preliminarily explore the molecular mechanism underlying IMF deposition in goats, and these observations may be useful for clarifying the role of *FATP4* in controlling intramuscular adipocytes in goats.

## 2. Materials and Methods

### 2.1. Ethics Statement

The Institutional Animal Care and Use Committee at Southwest Minzu University (Chengdu, China) examined and sanctioned every experimental procedure. Authorization number: S2020-013, updated in June 2004.

### 2.2. Preparation, In Vitro Culture and Cryogenic Preservation of Goat Intramuscular Preadipocytes

The isolation of primary intramuscular preadipocytes in goats was conducted using the method outlined earlier [19]. In detail, tissues from the longissimus dorsi muscles were sterilely obtained from two 2-day-old Jianzhou goats (chosen at random from two distinct two-day-old lambs), after being humanely euthanized through exsanguination. Tissues from the two goats were pooled and mixed in equal proportions prior to subsequent cell isolation procedures, which effectively resulted in a single biological source for all in vitro experiments. Following an equal mix, the tissue specimens underwent three washes with sterile phosphate-buffered saline enriched with 1% (*v*/*v*) penicillin–streptomycin solution, followed by slicing using sterile ophthalmic scissors. During the enzymatic breakdown, the tissue pieces were treated with 0.2% collagenase type II (Sigma-Aldrich, St. Louis, MO, USA) in a proportion of 1 mL of enzyme solution to 1 g of tissue, and softly agitated at 37 °C for a duration of 90 min.

To halt the enzymatic process, an identical amount of DMEM/F12 medium (Gibco, Beijing, China), enriched with 10% fetal bovine serum (FBS), was added. Following this, the acquired cell suspension underwent a sequential filtration process using sterile gauze and a 75-micron nylon mesh filter for cells. The filtrate was spun at a speed of 2000 rpm/min for 5 min at ambient temperature, then the supernatant was gently removed. Following this, the precipitated cells underwent a 5 min treatment with red blood cell lysis buffer, after which they were reconstituted in DMEM/F12 medium infused with 10% FBS and 1% penicillin–streptomycin dual-antibiotic. Subsequently, the cell mixture was moved to culture flasks measuring 25 cm^2^ and kept at a temperature of 37 °C in a moisture-rich environment with 5% CO_2_. The culture medium was substituted at 48 h intervals until the cells attained about 80% of their density of adhesion. The cells underwent a passaging process at a ratio of 1:3, reaching the third generation prior to their cultivation in 10 cm^2^ culture plates. For attaining adaptive differentiation, the growth medium was substituted with adipocyte differentiation induction medium. Adapted from the MEM/F12 medium, this variant comprised 10% fetal bovine serum, 1% penicillin–streptomycin dual-antibiotic, and 50 μmol/L oleic acid, supplied by SIGMA-ALDRICH in Tokyo, Japan. All subsequent functional experiments were performed with three technical replicates (*n* = 3) using cells derived from this pooled sample, with no independent biological replicates from individual goats.

### 2.3. Gene Cloning, Sequence Analysis and Construction of Phylogenetic Trees

This sequence was chosen for additional examination, guided by the anticipated sequence of the goat FATP4 gene listed in the GenBank database (accession number: XM_018055876.1). Targeted primers (forward primer: CGTTCCCTACTCTGCTAC; reverse primer: CTCTAGATCCAGTATCCGC) were crafted utilizing Primer Premier 5.0 software [20], and the *FATP4* gene sequence was cloned from goat subcutaneous adipocytes. Phylogenetic trees were constructed by building MEGA5 [21].

### 2.4. Construction of Expression Vectors, Chemical Synthesis of siRNA, and Subsequent Cell Transfection

The pcDNA3.1(+) plasmid was cut twice with Hind III and Bam HI, then ligated with the CDS region of the FATP4 gene, and named pcDNA3.1-*FATP4*. The empty pcDNA3.1(+) plasmid served as a negative control, designated as pcDNA3.1. Shanghai Gene Pharma Co., Ltd. (Shanghai, China) engineered and produced two siRNAs targeting the goat *FATP4* gene, named siRNA-FATP4-613 (CUGGACGACUACUCAAACATT) and siRNA-*FATP4*-1693 (GCGCCAACAACAAGAAGAUTT). Negative control (NC) was provided by the same company (Shanghai Gene Pharma Co., Ltd, Shanghai, China) (UUCUCCGAACGUGUCACGUTT).

The initiation of transfection occurred once the concentration of intramuscular preadipocytes in the 6-well plate escalated to 70–80%. This transfection method adheres to Lipofectamine ^TM^ 3000 (Carlsbad, CA, USA) guidelines, involving transfecting each of the six wells with either 1 μg of plasmid or 80 μM of siRNA.

### 2.5. Oil Red O Staining and Measurement of Intracellular Triglyceride Levels

Initially, the cells in the 6-well plate undergo three washes using PBS solution, followed by stabilization with a 4% formaldehyde solution at ambient temperature for half an hour. When performing the staining with Oil Red O, the working solution of Oil Red O (which is prepared by mixing 3 milliliters of a 5 g per-liter concentration of Oil Red O with isopropanol and 2 milliliters of deionized water) should be added to the cells, followed by incubation at room temperature for 20 min. Finally, the cells underwent a cleansing process using PBS and were imaged using a microscope. To measure Oil Red O levels, each well of the 6-well plate received 1 mL of isopropanol, and its absorbance value at 510 nm was determined using a spectrophotometer from Thermos Fisher Scientific (Shanghai, China).

The measurement of intracellular triglycerides was conducted using the Tissue Triglyceride (TG) Content Assay Kit (Applagin, E1013, Shanghai, China), adhering to the guidelines. First, the cells in the 6-well plate were cleansed thrice using PBS solution, followed by the addition of 200 microliters of triglyceride lysis buffer and a 10 min incubation on ice. Subsequently, the lysate was split into two segments, specifically for detecting BCA and triglycerides. Finally, the absorption of triglyceride was gauged at 550 nm, while the absorption of BCA was recorded at 562 nm. Utilizing the standard curve as a reference, the data were calculated, and the BCA values were used to normalize the triglyceride content.

### 2.6. Isolation of Total RNA and RT-qPCR Assay

According to the operation guidelines provided by the manufacturer, we extracted total RNA from the IMF cells of goats using RNAis Plus (Taro Company 9109, Kusatsu, Japan). RNA levels and purity were measured with the Nanodrop 2000 spectrophotometer (Thermos Fisher Scientific, Beijing, China), with the total RNA absorbance ratio (260/280 nanometers) ranging from 1.8 to 2.0. The reverse transcription kit (VA enzyme R323-01, Nanjing, China) was employed to transform 1 μg total RNA into cDNA. The Bio-Rad CFX96 PCR System was utilized for Real-Time PCR, employing Taq Pro Universal SYBR qPCR Master Mix (VA zyme, Q712-02, Nanjing, China) along with specific gene primers (Supplementary Table S1).

Ubiquitously expressed prefoldin-like chaperone (UXT) was chosen as the internal benchmark gene, and its comparative expression was determined through the 2^−∆∆CT^ method.

### 2.7. RNA Sequencing (RNA-Seq)

Intramuscular preadipocytes from goats were grown in 6-well plates, undergoing differentiation for a duration of 48 h. Cells were cleansed thrice using sterile PBS, followed by the addition of 1 mL Tizol, and then the samples were dispatched to Hangzhou LC-Bio Technologies (Hangzhou, China) for transcriptome sequencing (note: all cells were derived from pooled longissimus dorsi muscle tissues of two Jianzhou goats, with no independent biological replicates from individual animals; each group included three technical replicates). The gene expressions identified by RNA-seq are shown in Supplementary Table S2. Differential genes were screened using DESeq2 package, with the screening criteria set at *p* < 0.05 and fold change < 2 (Supplementary Table S3). To analyze gene enrichment, we utilized two databases: Gene Ontology (GO) (Supplementary Table S4) and Kyoto Encyclopedia of Genes and Genomes (KEGG) (Supplementary Table S5). We performed a GSEA analysis on the RNA-sequencing data, using goats as the standard for comparison.

### 2.8. CCK-8-Based Cell Proliferation Assay

The Cell Counting Kit-8 (CCK-8, AC11L054, Life-iLab, Shanghai, China) assay was utilized to evaluate the growth of intramuscular adipocytes. Cells were introduced into 96-well plates, ensuring three duplicate samples for every treatment. The test groups received transfections with pcDNA3.1-FATP4 or SI-FATP4, in that order, whereas the control groups underwent transfection with pcDNA3.1 or NC-FATP4. Following intervals of 0, 24, 36, and 48 h, each well received 10 μL of CCK-8 reagent (AC11L054, Life-iLab, Shanghai, China), and the cells underwent a 0.5 h incubation at 37 °C. Finally, absorbance levels were gauged with an enzyme-tagged device (Thermo Fisher Scientific, Carlsbad, CA, USA) at 450 nm wavelength.

### 2.9. Flow Cytometry for Apoptosis Analysis

To determine the proportion of cell apoptosis, we used the Annexin V-FITC/PI cell apoptosis detection kit (A211 VA Enzyme Company, Nanjing, China), and executed the experiment in accordance with the manufacturer’s instructions. Briefly, cells introduced into six-well plates underwent transfection via the identical procedure used in the CCK-8 experiments, followed by cell digestion and collection 48 h post-transfection. The gathered cell suspension was combined with 100 microliters of a 1× binding buffer. Ultimately, the cell mixture was supplemented with 5 microliters each of Annexin V-FITC and PI.

### 2.10. Western Blot Analysis

The extraction of cell proteins was performed with RIPA buffer (Solarbio Tech Inc., Beijing, China). Protease inhibitors (04693132001, Roche., Mannheim, Germany) and phosphatase inhibitors were introduced into the buffer to inhibit the breakdown of proteins. The total protein underwent separation through SDS-PAGE electrophoresis and was then moved to PVDF membranes for an in-depth analysis of abundance. For this study, the key antibodies used were: anti-β-actin (1:6000, BM0627, BOSTER, Wuhan, China), anti-p-AKT (1:2000, 4060, Cell Signaling Technology, Danvers, MA, USA), and anti-AKT (1:1000, ab32505, Abcam, Cambridge, UK). Target protein bands were visualized using an enhanced chemiluminescence (ECL) detection kit (Thermos Fisher Scientific, Waltham, MA, USA).

### 2.11. Data Analysis

Each experiment utilized three separate biological specimens. Utilizing SPSS version 22.0 (IBM Corp., Armonk, NY, USA), Student’s *t*-test or a one-way analysis of variance ANOVA was conducted. The significance was assessed using Duncan’s test, with values displayed as the mean plus or minus the standard error of the mean (SEM). Visualization of the data was performed with GraphPad Prism v8.0 (GraphPad Software, La Jolla, CA, USA), where ‘*’ denotes *p* < 0.05, ‘**’ denotes *p* < 0.01, and ‘***’ denotes *p* < 0.001.

## 3. Results

### 3.1. Cloning and Expression Pattern Analysis of Goat FATP4 Gene

**Figure 1:**
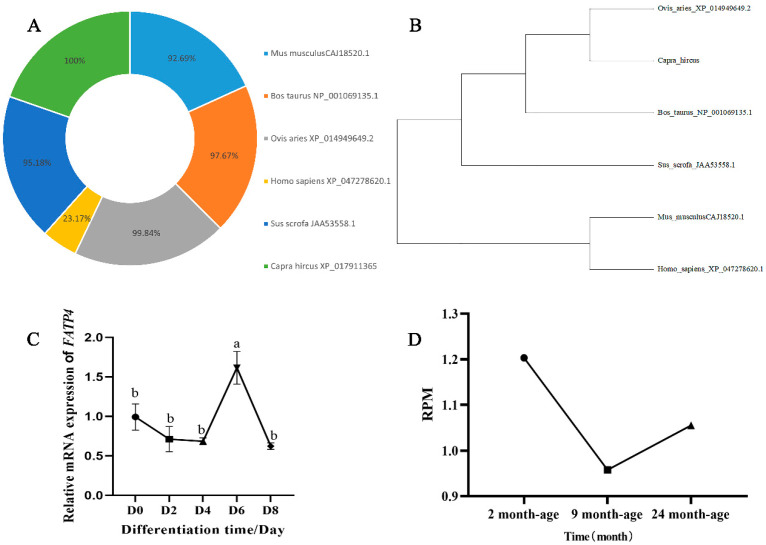
The sequence and expression analysis of *FAPT4* in goat. (**A**) The phylogenetic tree of *FATP4* amino acid sequences was developed using MEGA5.05. (**B**) *FATP4* amino acid similarity analysis. (**C**) Levels of *FATP4* mRNA observed on days 0, 2, 6, and 8 during the induced differentiation of intramuscular adipocytes (*n* = 3, technical replicates). (**D**) Expression of *FATP4* in three growth stages. All values are expressed as mean ± SEM; statistically significant differences are indicated by distinct letters.

Given the lack of validated sequences for the goat *FATP4* gene in the NCBI database, we achieved the first cloning of its mRNA sequence. The predicted sequence of capra hircus *FATP4* (GENBANK: XM018055876.1) was used to design primers (Table S1). A length of 2337 bp sequence was cloned, including a 1929 bp complete open reading frame (ORF) region sequence encoding 640 amino acid residues, with an ATG start codon and a TGA stop codon. The *FATP4* amino acid sequence comparison showed that goats showed the highest similarity of 99.84% with sheep, and 97.67%, 95.18%, 92.69%, and 23.17% with cattle, pigs, mice, and humans, respectively (Figure 1A). By amino acid phylogenetic tree analysis, goat *FATP4* is closely related to sheep, but distantly related to humans and mice (Figure 1B). During the goat preadipocytes differentiation, the mRNA expression levels of *FATP4* were continually reduced until the fourth day, and increased to the top at the sixth, followed by a sharp decrease at the eighth (Figure 1C). Our previous RNA-seq data [22] showed that the expression level of *FATP4* in 9-month-old goats was significantly lower than that in 2-month-old goats, and then it increased again in 24-month-old goats (Figure 1D).

### 3.2. Knockdown of FATP4 Contributes to Increased Lipid Deposition

**Figure 2:**
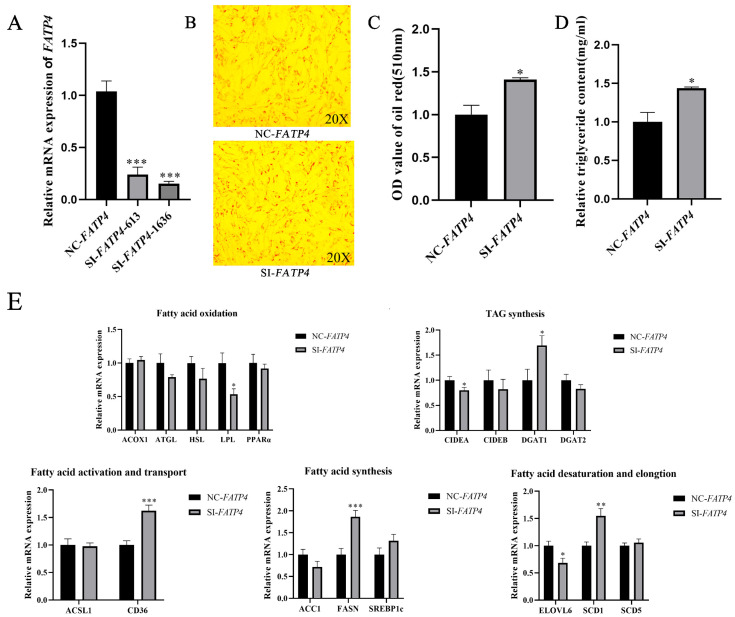
Knockdown of FATP4 contributes to increased lipid deposition. (**A**) RT-qPCR was used to detect the knockdown efficiency of *FATP4*. UXT was employed as the internal reference gene, with the negative control used for normalization. (**B**) The Oil Red O staining between control and siRNA treatment intramuscular adipocyte cells. (**C**) OD value of Oil Red O staining extraction after *FATP4* (SI-*FATP4*) interference. (**D**) Triglyceride content in *FATP4* knockdown cells. (**E**) Effect of *FATP4* knockdown on the expression of lipid metabolism-related genes. All values are expressed as mean ± SEM, ‘*’ denotes *p* < 0.05, ‘**’ denotes *p* < 0.01, and ‘***’ denotes *p* < 0.001.

To explore the regulatory role of *FATP4* in goat intramuscular lipid accumulation in vitro, we suppressed the expression of *FATP4* by siRNA treatment in cultured goat intramuscular preadipocytes. Both pairs of siRNAs (SI-*FATP4*-613 and SI-*FATP4*-1636) significantly reduced the mRNA expression of *FATP4* by 76% and 85%, respectively, relative to the control (*p* < 0.001, Figure 2A). For the following experiments, SI-*FATP4*-1636 was used for *FATP4* silencing. The relative content of TAG was significantly increased with the interference of *FATP4* (*p* < 0.05, Figure 2D), as well as the lipid droplet formation by Oil Red O staining (*p* < 0.05, Figure 2B,C).

Functional deficiency of *FATP4* significantly increased the expression of *CD36* (*p* = 0.0001), *SCD1* (*p* = 0.002), *FASN* (*p* = 0.0005), and DGAT1 (*p* = 0.031), and decreased the expression of *ELOVL6* (*p* = 0.017), *LPL* (*p* = 0.015), and *CIDEA* (*p* = 0.049) in this in vitro cell model. However, the expression of *SREBP1c*, PPARα, *SCD5*, ACSL1, *ACC1*, *ATGL*, *HSL*, *ACOX1*, *CIDEB* and *DGAT2* was not significantly affected (*p* > 0.05) by the suppression of *FATP4* (Figure 2E).

### 3.3. Overexpression of FATP4 Contributes to Inhibiting Lipid Deposition

**Figure 3:**
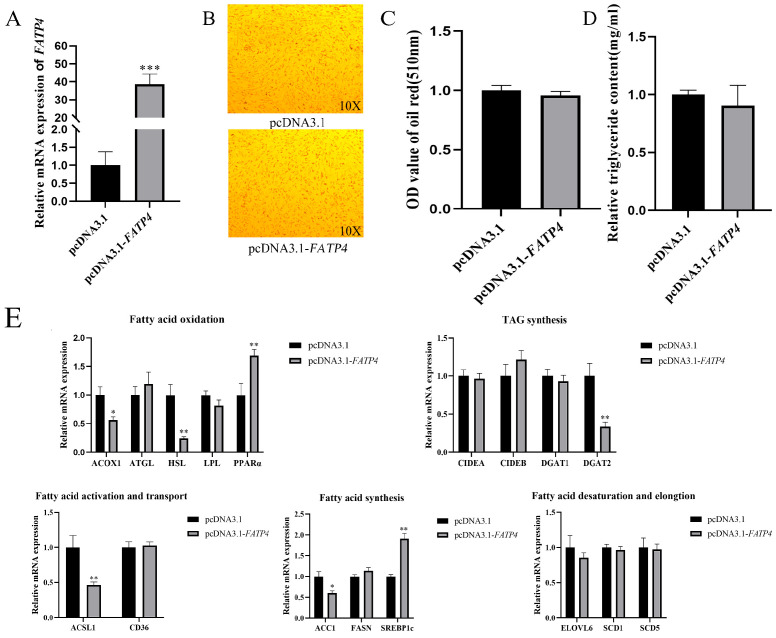
Overexpression of *FATP4* contributes to inhibiting lipid deposition. (**A**) Overexpression efficiency detection. UXT was employed as the internal reference gene, with the negative control used for normalization. (**B**) The Oil Red O staining between control and overexpression treatment intramuscular adipocyte cells. (**C**) OD value of Oil Red O staining extraction after *FATP4* (pcDNA3.1-*FATP4*) overexpression. (**D**) Triglyceride content in *FATP4* overexpression cells. (**E**) Effect of *FATP4* overexpression on the expression of lipid metabolism-related genes. All values are expressed as mean ± SEM, ‘*’ denotes *p* < 0.05, ‘**’ denotes *p* < 0.01, and ‘***’ denotes *p* < 0.001.

We constructed a pcDNA3.1-*FATP4* overexpression vector and upregulated its expression by 38-fold compared with the negative control group (pcDNA3.1) (*p* < 0.001, Figure 3A). The relative content of TAG did not significantly decrease following *FATP4* overexpression (Figure 3D). Similarly, the content of lipid droplets following *FATP4* overexpression was not significantly reduced by Oil Red O staining in this in vitro cell model (Figure 3B,C).

Meanwhile, to investigate changes in lipid metabolism-related genes induced by *FATP4* overexpression, we assessed their expression in *FATP4*-overexpressing cells in vitro. The results showed that overexpression of the *FATP4* gene did not significantly change *CD36*, *FASN*, *ATGL*, *LPL*, *ELOVL6*, *SCD1*, *SCD5*, *CIDEA*, *CIDEB,* and *DGAT1*, but significantly increased the expression levels of *ACSL1* (*p* = 0.0085), *SREBP1c* (*p* = 0.000008), *ACC1* (*p* = 0.0071), *ACOX1* (*p* = 0.012), *HSL* (*p* = 0.00084), *DGAT2* (*p* = 0.0017), and *PPARα* (*p* = 0.008) (Figure 3E).

### 3.4. Knockdown of FATP4 Inhibits Proliferation of Goat Intramuscular Preadipocytes In Vitro

**Figure 4:**
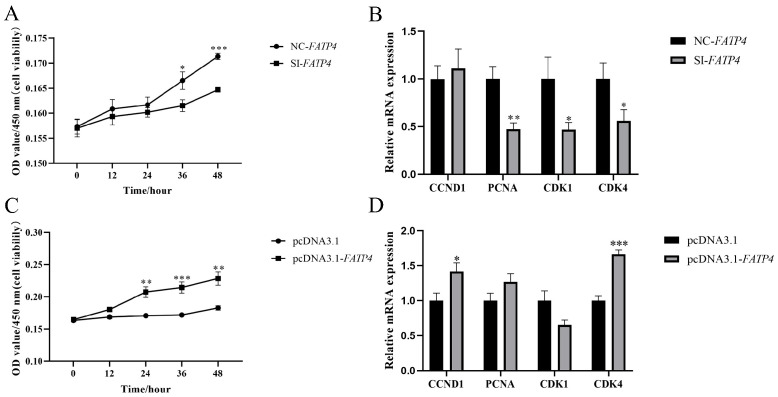
Effects of overexpression and interference with *FATP4* on adipocyte proliferation in cultured goat intramuscular preadipocytes in vitro. (**A**) *FATP4* downregulation inhibits goat adipocyte proliferation, as measured by 450 nm OD for cell viability. (**B**) Impact of knockdown of *FATP4* on the expression of cell proliferation-related genes. (**C**) Overexpression of *FATP4* promotes the cell proliferation of goat adipocytes, as measured by 450 nm OD for cell viability. (**D**) Impact of overexpression of *FATP4* on the expression of cell proliferation-related genes. All values are expressed as mean ± SEM, ‘*’ denotes *p* < 0.05, ‘**’ denotes *p* < 0.01, and ‘***’ denotes *p* < 0.001.

To characterize the role of *FATP4* in goat adipocyte proliferation in vitro, we utilized a CCK-8 assay and flow cytometry to evaluate goat adipocyte proliferation and apoptosis. The cell viability was significantly reduced (OD value of 450 nm) by the interference of *FATP4* compared with the control after the treatment of 36 h and 48 h (Figure 4A). Meanwhile, the downregulation of the *FATP4* gene significantly decreased the expression of *PCNA* (*p* = 0.0022), *CDK1* (*p* = 0.042) and *CDK4* (*p* = 0.047), but did not obviously affect the expression level of *CCND1* in this in vitro cell model (Figure 4B). Moreover, the downregulation of the *FATP4* gene decreased apoptosis in cultured goat adipocytes by flow cytometry assay (*p* < 0.05, Table S6A), and significantly reduced the mRNA expression levels of *Caspase7* (*p* = 0.0006), *Caspase3* (*p* = 0.000031), *Bax* (*p* = 0.00009) and *Bcl-2* (*p* < 0.01) mRNA (Table S6B).

### 3.5. Overexpression of FATP4 Promotes Proliferation of Goat Intramuscular Preadipocytes In Vitro

We employed the CCK-8 assay to assess the promotive effect of *FATP4* overexpression on cell proliferation. Our results demonstrated that cell viability was markedly elevated in the pcDNA3.1-*FATP4* group relative to the control group at 24 h (*p* < 0.01), 36 h (*p* < 0.001), and 48 h (*p* < 0.01) (Figure 4C). Consistent with these findings, the overexpression of *FATP4* significantly increased the expression levels of *CCND1* (*p* = 0.025) and *CDK4* (*p* = 0.000016), but did not affect the levels of *PCNA* and *CDK2* expression in this in vitro cell model (Figure 4D). At the same time, the overexpression of FATP4 significantly decreased apoptosis in cultured goat intramuscular preadipocytes (*p* < 0.05, Table S6C), and significantly decreased mRNA expression levels of *Caspase7* (*p* = 0.0099), *Bax* (*p* = 0.000015), and *Bcl-2* (*p* = 0.0015, Table S6D). However, the mRNA expression of *Caspase3* was not affected.

### 3.6. Knockdown of FATP4 Changes the Gene Expression Profile in Goat Preadipocytes In Vitro

**Figure 5:**
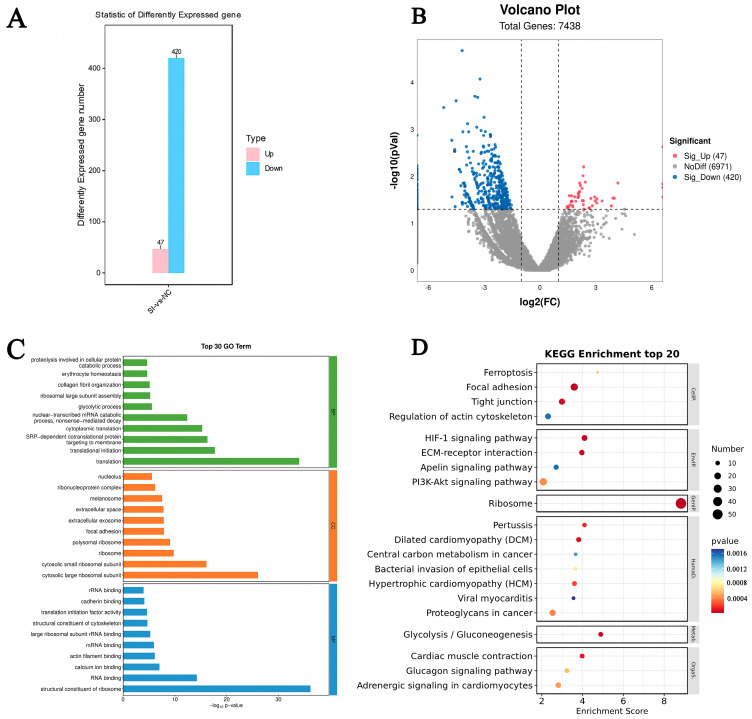
DEGs analysis after *FATP4* knockdown in cultured goat intramuscular preadipocytes. (**A**) Volcano plot of statistical differential genes, red indicates upregulated genes, blue indicates downregulated genes (*p* < 0.05, logFC > 2). (**B**) Bar graph of differential gene number, upregulated genes are marked in red, and downregulated genes in blue (*p* < 0.05, logFC > 2). (**C**) GO enrichment analysis of interfering *FATP4* genes. (**D**) Interference *FATP4* gene total differential gene KEGG analysis. Pathways in red boxes indicate later-studied pathways.

By RNA sequencing, a total of 467 differential genes were found in the *FATP4* knockdown group relative to control (*p* < 0.05, logFC > 2), of which 47 genes were upregulated, including the genes of *CTSK*, *BMP1*, and *CKAP2*, and 420 genes were downregulated, including the genes of *PRDX1*, *ANXA1*, *RPS6*, and *TXNRD1* (Figure 5A,B and Table S3), visualized in a heat map (Figure 5C). GO enrichment analysis revealed that the DEGs were mainly enriched in: (1) molecular functions (MF): structural constituent of ribosome, RNA binding, and calcium ion binding; (2) cellular components (CC): cytosolic large ribosomal subunit, cytosolic small ribosomal subunit, and ribosome; (3) biological processes (BP): translation, translational initiation and SRP-dependent co-translational protein targeting to membranes (Figure 5D and Table S4), including focal adhesion, HIF-1 signaling pathway, and PI3K-Akt signaling pathway (Table S5).

### 3.7. FATP4 Facilitates Lipid Deposition via the PI3K-Akt Signaling Pathway in Cultured Goat Adipocytes

**Figure 6:**
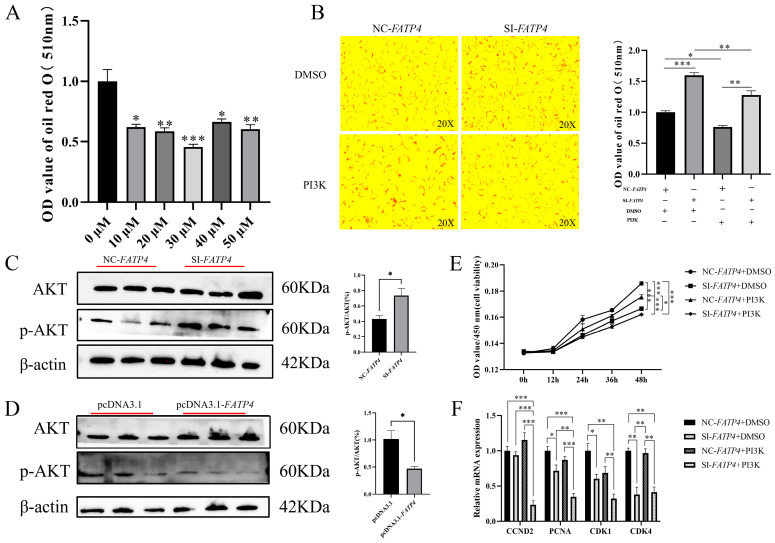
*FATP4* facilitates lipid deposition via the PI3K-Akt signaling pathway in cultured goat adipocytes. (**A**) The addition of different concentrations of PI3K pathway inhibitor (LY294002). (**B**) FATP4 interference-enhanced lipid deposition was dependent on PI3K signaling. (**C**) Changes in the levels of p-AKT/AKT proteins after interfering with *FATP4* detected by Western Blot. (**D**) Changes in the levels of p-AKT/AKT proteins after overexpression by Western Blot. (**E**) Interference with *FATP4* inhibits the proliferation of goat preadipocytes by synergizing with the PI3K signaling pathway, as measured by 450 nm OD for cell viability. (**F**) Effect of LY294002 after interfering with *FATP4* on the expression levels of cell proliferation-related genes. All values are expressed as mean ± SEM, ‘*’ denotes *p* < 0.05, ‘**’ denotes *p* < 0.01, and ‘***’ denotes *p* < 0.001.

KEGG enrichment analysis of DEGs from RNA-seq showed that the PI3K-Akt signaling pathway was significantly enriched. This result indicates that *FATP4* may regulate lipid deposition and proliferation through the PI3K-Akt signaling pathway in this in vitro cell model. To this end, we treated cells using different concentrations (0 μM, 10 μM, 20 μM, 30 μM, 40 μM, 50 μM) of Akt pathway inhibitor (LY294002), with DMSO as a control. The results showed that 30 μM of PI3K pathway inhibitor (LY294002) had the greatest effect on lipid deposition in cultured goat preadipocytes (Figure 6A). Therefore, 30 μM of PI3K pathway inhibitor (LY294002) was used in the following experiments. The results showed that the lipid deposition in goat preadipocytes was significantly inhibited by the combination use of FATP4 interference and LY294002 (Figure 6B). Moreover, p-AKT/AKT (%) was markedly decreased in the pcDNA3.1-*FATP4* group compared to in the pcDNA3.1 group (*p* < 0.05), and p-AKT/AKT (%) was markedly increased in the SI-*FATP4* group compared to in the NC group (*p* < 0.05) (Figure 6C,D). These results suggest that *FATP4* affects lipid deposition in goat preadipocytes by regulating the PI3K-Akt signaling pathway in vitro.

To confirm that the effect of *FATP4* on the proliferation of cultured goat preadipocytes are mediated by the PI3K-Akt signaling pathway, we added a PI3K pathway inhibitor (LY294002) along with the interference of *FATP4*. As shown in Figure 6E, treatment with the PI3K inhibitor (LY294002) significantly suppressed the proliferation of cultured goat preadipocytes in the NC-*FATP4*-PI3K group, compared to the NC-*FATP4*-DMSO control. Compared with the SI-*FATP4*-DMSO group, the SI-*FATP4*-PI3K group exhibited slightly reduced proliferation in cultured goat preadipocytes. In contrast, goat preadipocyte activity was significantly inhibited in the SI-*FATP4*-PI3K group compared to in the NC-*FATP4*-DMSO group, while interference with the *FATP4* gene together with LY294002 was found to exert an inhibitory effect on goat preadipocyte proliferation, consistent with the expression of proliferation-related genes (Figure 6F). These results suggest that the proliferation of goat preadipocytes is indeed partly influenced by the PI3K pathway in addition to the *FATP4* gene, and these observations reflect trends in technical replicates of pooled cell samples rather than generalizable effects in individual goats.

## 4. Discussion

Recent studies reveal that, contrary to earlier beliefs about its involvement in fatty acid transport across the plasma membrane, FATP4 is crucial for fatty acid absorption in several cell types [23,24,25]. According to Milger et al. [26], *FATP4* was shown to localize to the endoplasmic reticulum instead of the area surrounding the plasma membrane in many cell lines. It is known that endoplasmic reticulum membranes are embedded with proteins involved in phospholipid synthesis and distribution. In addition, *FATP4* has been shown to have acyl coenzyme A synthetase activity and a preference for LCFA [27]. However, the molecular mechanism underlying *FATP4*-mediated regulation of lipid metabolism and cell proliferation has not been fully elucidated. Our findings are novel in revealing the potential role of *FATP4* in shifting fatty acid fluxes toward cellular lipid synthesis or cell proliferation in cultured goat intramuscular preadipocytes, processes that may contribute to intramuscular fat (IMF) deposition with the involvement of the PI3K-Akt signaling pathway.

In this study, after cloning the goat *FATP4* gene, we used RNA silencing and overexpression techniques to explore the regulatory effects of *FATP4* on IMF deposition in vitro. Our results showed that silencing of *FATP4* significantly increased lipid deposition in goat intramuscular preadipocytes. In contrast, no significant differences were observed following *FATP4* overexpression, despite a tendency toward reduction. This is consistent with the findings of Herrmann et al. [27] in mice, where knockout mice showed a significant increase in lipid deposition despite not consuming more food. Meanwhile, Miner et al. [28] proposed that *FATP4* acts as an acyl coenzyme A synthase to convert fatty acids to fatty acyl coenzyme A, which is then translocated to mitochondria for β-oxidation. It is worth noting that the composition of IMF is primarily influenced by the quantity and dimensions of intramuscular adipocytes, of which the cell proliferation of intramuscular adipocytes [29] and apoptosis [30] are particularly important. Consistent with the results in Hs5578T and MDA-MB-231 cells [15], in the present study, silencing of *FATP4* inhibited cell proliferation in goat preadipocytes, while overexpression of *FATP4* in goat preadipocytes promoted cell proliferation. This is consistent with the finding of Lin et al. [18] that the epidermis of *FATP4* knockout mice showed hyperproliferation. These data may support the hypothesis that in our in vitro model, *FATP4* preferentially transfers fatty acids into goat preadipocytes for proliferation and lipid deposition. Specifically, knockdown of *FATP4* results in a blockage of the lipid transfer pathway for cell proliferation, which leads to triglyceride synthesis, whereas overexpression of *FATP4* promotes the lipid transfer pathway for cell proliferation but does not alter the original deposition of triglycerides.

Different from other FATP proteins known to mediate the cellular uptake of long-chain fatty acids [31,32,33], FATP4 is an intracellular enzyme located in the endoplasmic reticulum and predicted to mainly be involved in the transport of endogenous fatty acids [26]. Supporting the hypothesis, in the present study, the enhanced lipid synthesis induced by *FATP4* silencing significantly upregulated the expression of fatty acid synthase (*FASN*) and Diacylglycerol O-Acyltransferase 1 (*DGAT1*), the crucial enzymes for de novo fatty acid synthesis and cellular triglyceride [34,35], consistent with observations in intestinal cells of mice lacking calcium-independent phospholipase A2 (*IPLA*) in the VIA group [36]. The downregulated expression of *LPL* by *FATP4* silencing may also contribute to the accumulation of cellular triglyceride.

To elucidate the mechanism by which *FATP4* gene disruption promotes the accumulation of lipid droplets, we performed RNA-seq to analyze the changes in cellular mRNA levels after *FATP4* inhibition. Note that RNA-seq was performed on the pooled cell sample with three technical replicates, and thus, the differential expression results should be interpreted as preliminary mechanistic clues rather than definitive biological differences at the population level. The RNA-seq results indicate the involvement of the PI3K-AKT signaling pathway in mediating cellular lipid deposition in the absence of *FATP4*. As a crucial intracellular signaling route, the PI3K-Akt signaling pathway plays a vital role in facilitating cell proliferation and inhibiting apoptotic cell death [37]. AKT, also named protein kinase B, as the primary downstream PI3K signaling molecule, widely regulates cell division, differentiation, migration, and glucose metabolism by phosphorylating its downstream target proteins [38], and it has been well studied in follicular Theca cells [39] and Sertoli cells [40] in goat. In the present study, we conducted functional rescue experiments in goat intramuscular preadipocytes to further verify the involvement of the PI3K-AKT signaling pathway. The inhibition of the PI3K-AKT pathway by LY294002, a synthetic small molecule that serves as a specific PI3K inhibitor by competitively binding to the ATP-binding pocket of the kinase. Our results showed that the addition of PI3K pathway inhibitor (LY294002) inhibited the proliferation of goat preadipocytes. Bakin et al. [41] demonstrated that LY294002 inhibits cell proliferation primarily through activation of AMPKα1 and inactivation of AKT. Wang et al. [42] also demonstrated that LY294002 alone significantly inhibited the proliferation of PK59 and KLM1-R cells. In addition, the treatment of LY294002 canceled the triglyceride accumulation and enhanced the inhibition on cell proliferation, despite non-significance, induced by *FATP4* silencing. These data may provide a fact that the activation of PI3K-AKT is essential for the utilization of cellular fatty acids toward both cell proliferation and lipid synthesis in an in vitro model.

We also realized the limitation of the lack of a specific antibody for goat *FATP4* protein detection. Actually, we tried several commercial antibodies targeting other species, including bovine, rabbits, mice and so on. None of the antibodies was suitable for goat *FATP4* detection. Meanwhile, due to limitations of experimental conditions and animal sources, cells were isolated from pooled tissues of two goats, and all subsequent in vitro experiments were performed with technical replicates only (*n* = 3), without independent biological replication. In the present study, we detected the protein expression of AKT and phosphorylated AKT, which partly proved that the treatment of *FATP4* indeed worked in cultured goat preadipocytes. An enhanced result may be validated using a goat-specific *FATP4* antibody for future study.

Beyond the above, several additional limitations should be acknowledged. First, this study was performed at the cellular level in vitro, and the results may not fully reflect the physiological status in vivo. Second, only a small number of animals were used for primary preadipocyte isolation, which may restrict the representation of biological variability to some extent. Third, the regulatory function of *FATP4* needs to be further verified by in vivo experiments. Finally, the effects observed in intramuscular preadipocytes may be tissue-specific, and whether similar regulatory mechanisms exist in other tissues remains to be explored.

## 5. Conclusions

**Figure 7:**
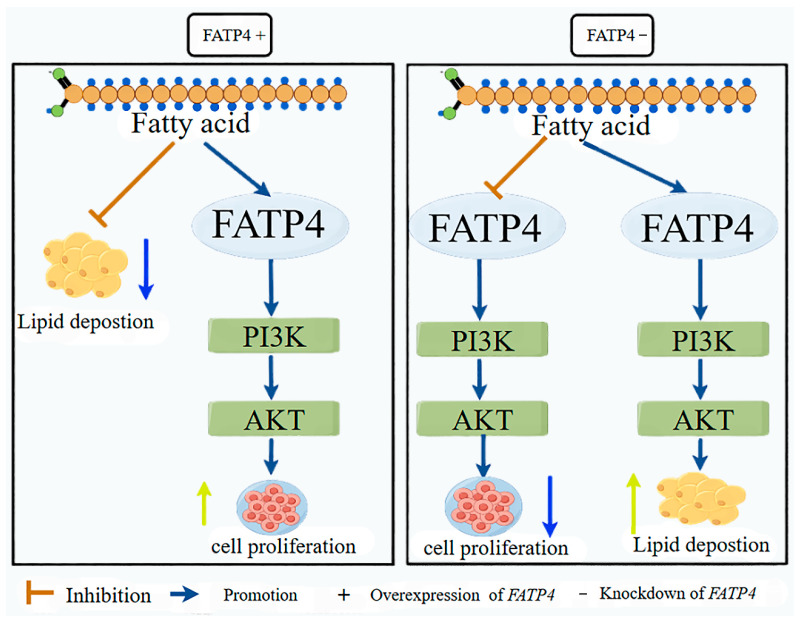
*FATP4* fatty acid distribution pattern diagram. (**Note:** The yellow arrow indicates upregulation, and the light blue arrow indicates downregulation).

We firstly obtained the sequence of the *FATP4* gene by in vitro cloning and demonstrated that *FATP4* may function as the fatty acid donor for cell proliferation priority with the activity of PI3K-AKT pathway in cultured goat intramuscular. Its functional deficiency may result in the endogenous fatty acid utilization change toward lipid synthesis in this in vitro model. These results provide novel insights into the function of the FATP4 gene in cellular lipid metabolism, and help clarify its potential regulatory mechanism during goat intramuscular fat deposition (Figure 7).

While these findings offer preliminary molecular clues that could inform future goat breeding research aimed at improving meat quality, the in vitro nature of the study and lack of biological replication mean that direct application to marker-assisted selection (MAS) requires further in vivo validation in larger populations. The identified role of FATP4 in regulating intramuscular fat deposition in cultured cells may lay a foundation for subsequent studies targeting lipid metabolism traits in goats.

For further research directions, in vivo validation experiments using larger cohorts of goats are required to confirm the physiological function of FATP4 observed in this in vitro model. Additionally, the development of specific FATP4 activators or inhibitors would offer potential tools to manipulate lipid metabolism in livestock, while exploring tissue-specific regulatory mechanisms of FATP4 across different adipose depots will help to refine targeted strategies for optimizing meat quality traits in goat production.

## Data Availability

The datasets from the present study can be accessed via the NCBI Bio Project database (https://www.ncbi.nlm.nih.gov/bioproject, accessed on 6 March 2025), mRNA-Seq accession numbers are PRJNA1080472.

## Supplementary Materials

The following supporting information can be downloaded at: https://www.mdpi.com/article/10.3390/ani16081129/s1, Table S1: Primers for quantitative real-time PCR (RT-qPCR), Table S2: ALL_FPKM, Table S3: Differential genes were screened using DESeq2at *p* < 0.05 and fold change < 2, Table S4: GO enrichment analysis, Table S5: KEGG enrichment analysis, Table S6: Effect of *FATP4* on the apoptosis of goat adipocytes.
